# Omics data integration suggests a potential idiopathic Parkinson’s disease signature

**DOI:** 10.1038/s42003-023-05548-w

**Published:** 2023-11-20

**Authors:** Alise Zagare, German Preciat, Sarah. L. Nickels, Xi Luo, Anna S. Monzel, Gemma Gomez-Giro, Graham Robertson, Christian Jaeger, Jafar Sharif, Haruhiko Koseki, Nico J. Diederich, Enrico Glaab, Ronan M. T. Fleming, Jens C. Schwamborn

**Affiliations:** 1 Luxembourg Centre for Systems Biomedicine (LCSB), University of Luxembourg, 7, Avenue des Hauts-Fourneaux, 4362 Esch-sur-Alzette, Luxembourg; 2 Metabolomics and Analytics Center, Leiden Academic Centre for Drug Research, Leiden University, 2300 RA Leiden, The Netherlands; 3 School of Medicine, University of Galway, University Rd, Galway, Ireland; 4 Laboratory for Developmental Genetics, RIKEN Center for Integrative Medical Sciences (IMS), Kanagawa, 230-0045 Japan; 5 Centre Hospitalier de Luxembourg (CHL), 4, Rue Nicolas Ernest Barblé, L-1210 Luxembourg, Luxembourg

**Keywords:** Parkinson's disease, Metabolic engineering

## Abstract

The vast majority of Parkinson’s disease cases are idiopathic. Unclear etiology and multifactorial nature complicate the comprehension of disease pathogenesis. Identification of early transcriptomic and metabolic alterations consistent across different idiopathic Parkinson’s disease (IPD) patients might reveal the potential basis of increased dopaminergic neuron vulnerability and primary disease mechanisms. In this study, we combine systems biology and data integration approaches to identify differences in transcriptomic and metabolic signatures between IPD patient and healthy individual-derived midbrain neural precursor cells. Characterization of gene expression and metabolic modeling reveal pyruvate, several amino acid and lipid metabolism as the most dysregulated metabolic pathways in IPD neural precursors. Furthermore, we show that IPD neural precursors endure mitochondrial metabolism impairment and a reduced total NAD pool. Accordingly, we show that treatment with NAD precursors increases ATP yield hence demonstrating a potential to rescue early IPD-associated metabolic changes.

## Abstract (web summary)

Transcriptomics and metabolomics data integration coupled with systems biology reveal signature differences in idiopathic Parkinson’s disease vs healthy controls, with pyruvate, amino acid, lipid and NAD metabolism as most dysregulated pathways.

## Introduction

Parkinson’s disease (PD) is a neurodegenerative disorder with increased prevalence among elderly people. The main cellular hallmarks of PD, such as selective loss of dopaminergic neurons and the presence of Lewy bodies in the PD patient’s brain, have been well described; however, disease molecular mechanisms are not yet clear. Moreover, approximately 90% of all PD cases are classified as sporadic or idiopathic^1^. Investigation of idiopathic PD (IPD) cases is challenging due to unclear etiology and their multifactorial nature^1,2^. One of the explanations of how the interaction between multiple factors contributes to PD development is proposed by the “multiple hit” theory^3,4^. It suggests that the risk to develop PD is already determined at the early neurodevelopmental stage, where the ‘first hit’ in the form of genetic mutation, exhausts compensatory mechanisms, modulating cell resistance to perturbations of cellular homeostasis. The “second hit” as environmental or lifestyle factors later leads to the disease onset and determines its progression.

Changed cellular metabolism as a response to the ‘first hit’ might eventually lead to increased dopaminergic neuron vulnerability and susceptibility to PD-related neurodegeneration. Lately, the role of metabolic dysregulation has been highlighted in the context of neurodegenerative diseases^5–9^. Moreover, diminished mitochondrial energy production capacity is among the most discussed pathogenic mechanisms implicated in both familial and idiopathic forms of PD development^10–13^. In addition to the deficient energy metabolism, dysregulation in lipid metabolism has also been linked to PD development^14–18^. Furthermore, other studies describe impaired cofactor metabolism contribution to neurodegeneration and amino acid concentration changes, the latter also having been proposed as biomarkers of PD progression^19–22^.

In this study, we were interested in the initial metabolic changes occurring even before neuronal differentiation, to understand the basis of dopaminergic neuron vulnerability. Investigation of early metabolic changes might provide a deeper understanding of IPD pathogenesis and provide clues to disease-modifying strategies. We used iPSCs-derived neuroepithelial stem cells (NESCs), which are pre-patterned to midbrain/hindbrain identity and can give rise to neurons, including midbrain dopaminergic neurons, as well as to oligodendrocytes and astrocytes^23–27^. We analyzed NESCs from three IPD patients and three healthy individuals at the transcriptomics and metabolomics levels to obtain an overview of the IPD-associated metabolic changes at the very early neurodevelopment stage. In addition, we used the XomicsToModel pipeline^28,29^ to extract context-specific models from the most comprehensive genome-scale human metabolic network Recon 3D^30^. The flux distribution was further addressed using entropic flux balance analysis. Additionally, predicted metabolic alterations we compared to PD-associated metabolic biomarkers reported in clinical studies.

We here show that IPD NESCs have changed pyruvate, lipid and amino acid metabolism. In addition, we demonstrate that IPD NESCs have decreased mitochondrial respiratory capacity and a NAD pool deficiency. Moreover, using NAD precursors, we show rescue of decreased levels of ATP in IPD NESCs, highlighting role of NAD metabolism in energy generation sustainability, and as a preventative PD therapeutic target. Finally, using data integration analysis, we confirm the link between energy generation, lipid metabolism and NAD regeneration, which leads to the identification of glycerol-3-phosphate as the main intermediate of metabolic pathways altered in IPD NESCs.

## Results

### Transcriptomic and metabolic profiles reveal neurodevelopmental and metabolic alterations in IPD neural precursor cells

**Fig. 1:**
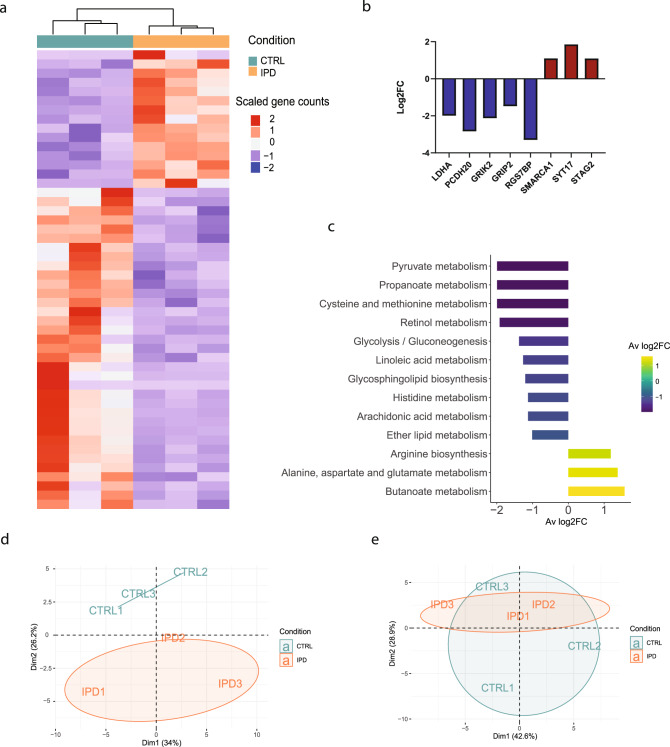
Transcriptomic and metabolic profiles reveal neurodevelopmental and metabolic alterations in IPD neural precursor cells. **a** Heatmap of scaled gene counts of significantly differentially expressed genes (*p* < 0.05) between IPD and control NESCs. **b** Log2FC of the top significantly expressed genes (FDR < 0.05) displaying gene expression difference in IPD NESCs compared to control cells. **c** The most dysregulated metabolic pathways, selected by the average of Log2FC < −1 or >1 of genes annotated in each pathway. The color represents the Log2FC. **d** Principal component scores plot of non-polar metabolites detected by untargeted GC-MS analysis. **e** Principal component scores plot of polar metabolites detected by untargeted GC-MS analysis.

First, we compared transcriptomic and metabolic differences between three female IPD patients and three age-gender matched control NESC lines (Supplementary Fig. 1a, b). We identified 678 significantly differentially expressed genes (DEGs) (*p* < 0.05), showing a strong transcriptomic difference between IPD and control NESCs (Fig. 1a, Supplementary Data 1). The top significant DEGs (FDR < 0.05) included the key regulator of the final step of glycolysis—lactate dehydrogenase A (*LDHA*), being nearly two-fold downregulated in IPD NESCs (Fig. 1b). Furthermore, all other most significant DEGs (*PCDH20, GRIK2, GRIP2, RGS7BP, SMARCA1, SYT17, STAG2*) are known to be involved in neurodevelopmental processes. Overall, a gene ontology enrichment analysis showed that DEGs are involved in the regulation of cell development, cell cycle, synapse membrane potential, as well as metabolic processes particularly related to lipid metabolism (glycerolipid and phospholipid biosynthetic processes, phosphatidylinositol metabolic process, lipase, and phospholipase activity) and cofactor metabolic processes (Supplementary Fig. 2a). Next, we functionally annotated DEGs using the KEGG database for gene set enrichment analysis^31^. We found that 286 unique pathways were associated with these DEGs. ‘Metabolic pathways’ was the most enriched functional term (Supplementary Fig. 2b). Consistent with the most significant DEGs, relation to neurodevelopment, neuroactive-ligand receptor interaction and neurodegeneration pathways were also found in the top 10 of the most enriched KEGG terms (Supplementary Fig. 2b). The negative average log2 fold change (FC) of DEGs annotated to metabolic pathways, indicated a general negative regulation of metabolic processes in IPD NESCs. As the most dysregulated metabolic pathways, we selected the ones with the respective log2FC below −1 or above 1. Following the observed two-fold downregulation of *LDHA*, the lowest log2FC was detected for pyruvate metabolism (Fig. 1c). Similarly a negative, nearly two-fold log2FC was found for propanoate metabolism and cysteine and methionine metabolism, while the highest log2FC of 1.6 was for butanoate metabolism suggesting a particular role of short-fatty acid metabolism in IPD NESCs. Furthermore, genes associated with lipid metabolism-related pathways—linoleic acid, glycosphingolipid and ether lipid metabolism also had a negative log2FC.

Next, we performed an untargeted gas chromatography-mass spectrometry (GC-MS) analysis of the polar and non-polar-phase intracellular metabolites. A principal component analysis (PCA) showed a strong separation between IPD and control samples for non-polar metabolites further suggesting alterations in lipid metabolism in IPD NESCs (Fig. 1d). In contrast, there was no clear separation between IPD and control sample groups based on polar metabolite abundances, mainly because of the high variability in the metabolic profiles between control NESCs (Fig. 1e). However, the close proximity of all three IPD samples in the PCA plot implies a similarity among the IPD metabolic profiles, suggesting a disease-dependent metabolic signature.

### IPD neural precursors show reduced ability to metabolize various mitochondrial substrates and impaired mitochondrial respiratory capacity

**Fig. 2:**
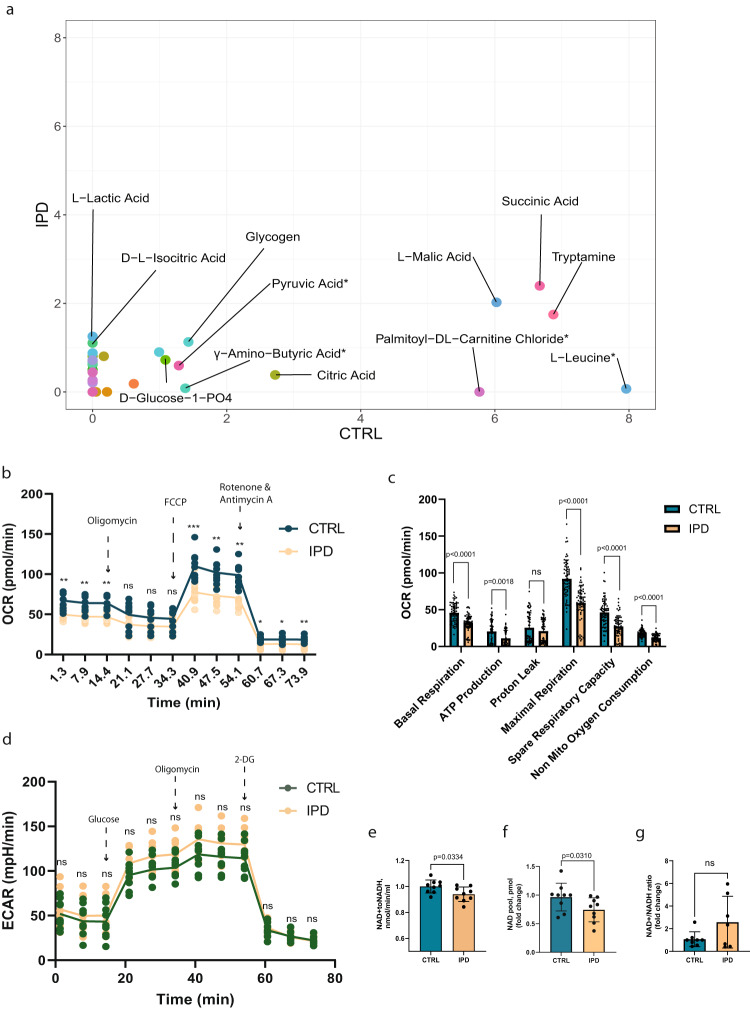
IPD neural precursors show reduced ability to metabolize various metabolic substrates and impaired mitochondrial respiratory capacity. **a** Scatter plot of the maximal metabolic rate of different substrates. Each dot represents a unique substrate placed in the plot according to the metabolic rate by which it has been metabolized by the IPD (*y*-axis) and control (*x*-axis) NESCs. The maximal metabolic rate is normalized by background subtraction and cell density in the respective well. The median rate between the three lines of each condition is considered. Substrates metabolized with the normalized maximal rate above 1 are labeled. **b** Oxygen consumption rate (OCR) over time representing mitochondrial respiratory capacity. Basal respiration is measured until the injection of oligomycin which inhibits complex V activity, resulting in a decrease in respiration, which is linked to ATP production. FCCP injection disrupts ATP synthesis and mitochondrial membrane potential, allowing measurement of maximal respiration and spare respiratory capacity. The final injection is a mixture of complex I and complex III inhibitors—rotenone and antimycin A. Here mitochondrial respiration is shut down, enabling the calculation of nonmitochondrial respiration. Statistics: unpaired *t*-test. Significance asterisks represent *P < 0.05, **P < 0.01, ***P < 0.001. Error bars represent mean + SD. *N* = 3 independent experiments. **c** Bar graphs of mitochondrial respiratory capacity features. Statistics: non-parametric Mann-Whitney test. Error bars represent mean + SD. *N* = 3 independent experiments, each data point represents a measurement of a single well of the assay. **d** Extracellular acidification rate (ECAR) over time representing glycolytic function. Before glucose injection, ECAR shows non-glycolytic acidification caused by processes in the cell other than glycolysis. The first injection of glucose enables measurement of the rate of glycolysis under basal conditions. The second injection of oligomycin, a complex V inhibitor, enhances the energy production via glycolysis, revealing the maximum glycolytic capacity. The final injection of 2-deoxy-glucose (2-DG), a glucose analog that inhibits glycolysis, allowing to measure glycolytic reserve. *N* = 3 independent experiments. **e** Pyruvate dehydrogenase activity measured based on nmol of generated NADH from NAD+ over time, which is proportional to enzyme activity. Statistics: non-parametric Mann-Whitney test. Error bars represent mean + SD. *N* = 3 independent experiments. **f** Total pool of NAD (NAD+ and NADH) concentration in pmol relative to the control samples. Statistics: non-parametric Mann-Whitney test. Error bars represent mean + SD. *N* = 3 independent experiments. **g** NAD+/NADH ratio relative to the control samples. Statistics: non-parametric Mann-Whitney test. Error bars represent mean + SD. *N* = 3 independent experiments.

The negative regulation of several metabolic processes and the downregulation of *LDHA* in IPD NESCs suggest a dysregulation of ATP-producing pathways. Therefore, we wanted to investigate the mitochondrial capacity to metabolize various metabolic substrates and the ability to generate ATP. First, we assessed mitochondrial functionality using MitoPlateS1, which is a microplate pre-coated with various NADH and FADH2-producing metabolic substrates^32,33^. We observed that the variety of substrates metabolized by IPD NESCs is reduced (Fig. 2a, Supplementary Fig. 3). The highest maximal metabolic rate for IPD NESCs was observed for succinic acid, malic acid and tryptamine. However, the metabolic rate for these substrates was about two times lower in IPD NESCs compared to control NESCs. Cytoplasmic substrates, particularly, metabolites of glycogenolysis, such as glycogen and glucose-1-PO_4_ were metabolized at similar rates by IPD and control NESCs. For other substrates, such as r γ-amino-butyric acid (GABA), palmitoyl DL-carnitine chloride and L-leucine the maximal metabolic rate was between 1.5 and 8 times higher for control NESCs compared to IPD NESCs.

Next, we looked more specifically at mitochondrial respiration efficiency using the Seahorse Extracellular Flux Analyzer. IPD NESCs demonstrated a significant decrease in all parameters of the mitochondrial stress test (Fig. 2b, c). Already basal respiration was detected in significantly reduced levels. After the addition of oligomycin, which is an inhibitor of ATP synthase, we observed significantly decreased ATP levels. Finally, after the addition of mitochondrial oxidative respiration uncoupler—carbonyl cyanide-p-trifluoromethoxyphenylhydrazone (FCCP), we detected that maximal respiratory capacity is decreased as well. Furthermore, we analyzed glycolysis efficiency using the same Seahorse approach (Fig. 2d). After the addition of glucose, IPD NESCs showed the same rate of glucose conversion to pyruvate as control cells. Subsequent addition of oligomycin to detect the maximum glycolysis capacity also did not show a significant difference between IPD and control NESCs. Furthermore, the glycolytic reserve was also equal between the conditions, indicating that glycolysis is neither impaired nor significantly overactivated in IPD NESCs.

Since we observed impairment in mitochondrial metabolism but not in glycolysis, we examined the activity of pyruvate dehydrogenase (PDH), which is at a transition step between glycolysis and the TCA cycle. By, using NAD+ reduction to NADH as a readout, we indeed found that the activity of PDH was significantly downregulated in IPD NESCs (Fig. 2e). However, neither the protein levels of the PDH major subunit E1 alpha (PDHA1) nor the mRNA levels were significantly affected (Supplementary Fig. 4a–c). Together this indicates an impaired connection between glycolysis and mitochondrial metabolism potentially due to the decreased levels of NAD. To further investigate this, we measured the concentration of total NAD. We confirmed that the NAD pool was decreased in IPD NESCs (Fig. 2f), however, the NAD+/NADH was not altered significantly (Fig. 2g). Since NAD is an essential cofactor in many metabolic reactions, its deficiency would explain not only the impaired activity of PDH but also the limited variability of mitochondrial substrate metabolism and decreased respiration capacity of IPD neural precursors.

### Metabolic modeling reveals cholesterol and tyrosine metabolism as the most dysregulated metabolic pathways in IPD and confirms changed NAD metabolism

In addition to the dysregulation of lipid, pyruvate and several amino acid metabolisms as suggested by RNA sequencing and untargeted metabolomics, we found that the NAD amount is reduced in IPD NESCs. To further investigate the overall metabolic differences between IPD and control NESCs, and to confirm the most dysregulated pathways, we complemented our analysis with a constraint-based modeling approach. Metabolic models were generated using XomicToModel pipeline^28^. Using Recon 3D as a reference of human metabolism, we extracted a unique context-specific metabolic model for each IPD and control cell line and further constrained models using bibliomic constraints regarding the dopaminergic neuron metabolism and cell culture media composition^29,34–36^. First, we performed a topological composition analysis of the generated models by correlating genes, reactions and metabolites present in each model (Supplementary Fig. 5a–c). Overall, we did not observe a strong correlation pattern between model composition (ρ_Av_ (genes) = 0.5; ρ_Av_ (reactions) = 0.4; ρ_Av_ (metabolites) = 0.45) of IPD models nor control models, suggesting that each model represents a distinct, disease independent metabolic network.

**Fig. 3:**
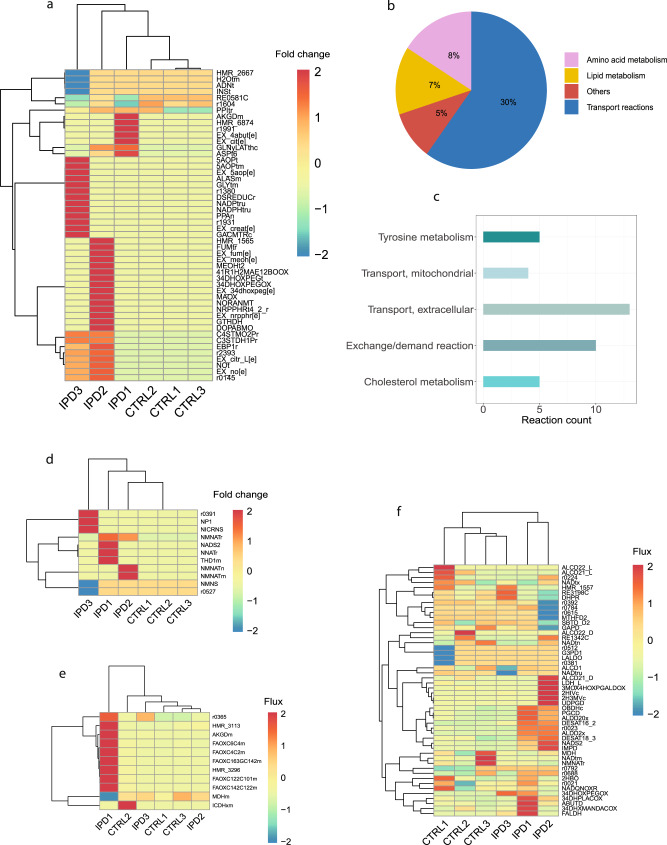
Metabolic modeling. **a** Unsupervised clustering of the top 50 reactions with the greatest fold change of flux relative to the mean flux of control models. Flux distribution was determined using eFBA. **b** Metabolic pathways to which the top 50 reactions are assigned, demonstrating the percentage of the top 50 reactions belonging to each pathway. **c** The top five subsystems with the highest reaction count were determined by assigning the top 50 most changed reactions to the Recon 3D subsystems. **d** Unsupervised clustering of NAD+ metabolism reactions based on the fold change of flux relative to the mean flux of control models. Flux distribution was determined using eFBA. **e** Unsupervised clustering of estimated flux for the NAD+ involving reactions in the mitochondria. Flux distribution was determined using eFBA. **f** Unsupervised clustering of estimated flux for the NAD+ involving reactions in the cytosol. Flux distribution was determined using eFBA.

A mathematical modeling approach maximizing the entropy of forward and reverse fluxes was employed for predicting reaction fluxes within each generated model. The approach uses entropic flux balance analysis (eFBA) to avoid thermodynamically infeasible cycles in the metabolic network, being in accordance with the first law of thermodynamics. The top 50 reactions with the highest absolute FC for all models relative to the mean flux of control models, separated IPD and control models using unsupervised clustering (Fig. 3a, Supplementary Data 2). Control models showed a similar flux pattern for the identified reactions, while IPD models demonstrated a model-specific, distinct pattern of flux change for the same reactions. We classified these top 50 reactions into metabolic pathways, such as transport reactions, lipid metabolism and amino acid metabolism. Subsystems that only had one reaction assigned, we defined as ‘others’ (Fig. 3b). Further, we identified to which Recon 3D subsystems these reactions belong. The top five subsystems from the defined metabolic pathways with the highest reaction number assigned were Cholesterol metabolism, Tyrosine metabolism, Mitochondrial transport, Extracellular Transport and Exchange/demand reactions (Fig. 3c, Supplementary Data 2).

Next, we wanted to confirm altered NAD metabolism in IPD NESCs. We analyzed the metabolic flux of reactions that belong to the NAD metabolism subsystem in Recon 3D (Fig. 3d, Supplementary Data 3). This subsystem mostly includes reactions involved in NAD synthesis. We observed that IPD and control models clustered separately due to the increase of flux in IPD models relative to the control models. Additionally, we investigated the flux distribution for the reactions where NAD is involved in mitochondria or the cytosol (Fig. 3e, f, Supplementary Data 4 and 5). We observed a strong flux increase for one of the IPD models for the fatty acid oxidation reactions, consuming NAD in the mitochondria, while there was no strong flux difference for other reactions in any other IPD NESC model. However, we noticed that there is a separation between IPD and control models for the cytosolic NAD reactions. There the flux pattern was also rather increased in IPD models compared to the control models.

### Glycerol-3-phosphate links altered pathways in IPD neural precursor cells

**Fig. 4:**
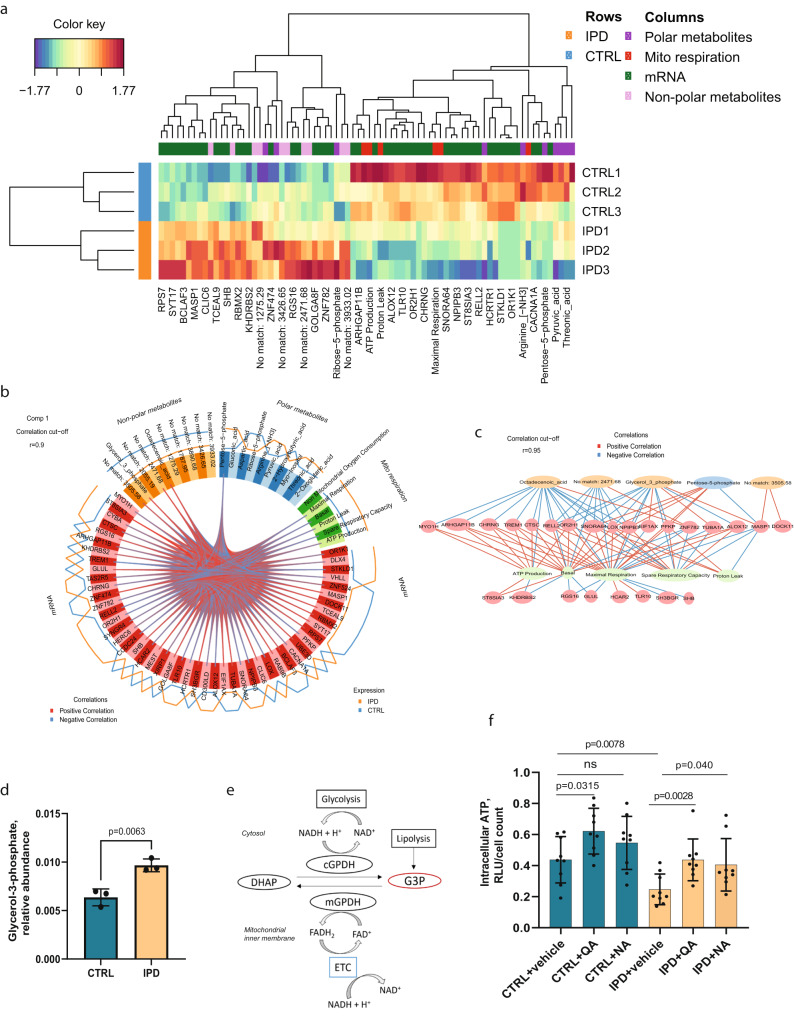
Data integration. **a** Heatmap of the top discriminant features between the IPD and control NESCs. **b** Circos plot showing the correlation between features contributing to the variation of the component 1. Correlation threshold: r = 0.9. **c** Correlation network of features contributing to the variation of the component 1. Correlation threshold: r = 0.95. **d** Relative abundance of glycerol-3-phosphate. Statistics: Welch’s *t*-test. Error bars represent mean + SD. *N* = 3 biologically independent samples. **e** Graphical representation of glycerol-3-phosphate (G3P) as an intermediate metabolite in glycolysis, lipid metabolism and oxidative phosphorylation and its role in NAD metabolism. G3P synthesis from dihydroxyacetone phosphate (DHAP) by cytosolic glycerol-3-phosphate dehydrogenase (cGPDH) regenerates cytosolic NAD+ from the NADH that is generated by glyceraldehyde-3-phosphate dehydrogenase in glycolysis. The G3P shuttle also facilitates electron transport between cytosol to mitochondria. Flavin linked mitochondrial glycerol-3-phosphate dehydrogenase (mGPDH) oxidases G3P at the same time reducing flavin adenine dinucleotide (FAD) to FADH_2_ and transferring electrons to ubiquinone pool of the electron transport chain (ETC)_._ ETC oxidazes NADH generated in TCA to replenish the mitochondrial NAD+ pool. G3P can also be produced from glycerol, which is the end product of lipolysis. **f** Intracellular ATP levels measured in relative light units (RLU) and normalized to the cell number in samples treated with vehicle, 20 nM quinolinic acid (QA) and 5 mM nicotinic acid (NA). Statistics: non-parametric Mann-Whitney test. Error bars represent mean + SD. *N* = 3 independent experiments.

We applied a supervised data integration approach to transcriptomics, metabolomics and Seahorse mitochondrial assay data to investigate a potential link between dysregulated metabolic pathways. The top 10 polar and non-polar metabolites, the top 50 gene transcripts and all six mitochondrial respiration features measured by the Seahorse assay were integrated. We applied a Partial Least Squares discriminant analysis (PLS-DA) to these data features to assess the separability between disease states of NESCs—IPD or healthy control. All four datasets showed a separation between sample conditions along the first component (Supplementary Fig. 6). The top discriminative features after merging the datasets are shown in Fig. 4a. Next, we looked at the correlation between features contributing to the variance captured by the first component (Fig. 4b). We observed a strong correlation (r = 0.9) between all genes, four non-polar metabolites, one polar metabolite and five mitochondrial respiration measurements. Hence, we decided to increase the correlation threshold to r = 0.95 in order to reduce the network (Fig. 4c). We observed that non-polar metabolites mostly had a negative correlation with genes, while pentose-5-phosphate, the only polar metabolite in the network, showed a positive correlation with several genes that further demonstrated a positive correlation with the mitochondrial respiration. Further, we looked at GC-MS datasets to evaluate whether any of these metabolites present in the correlation network are significantly differentially abundant between IPD and control samples. We identified glycerol-3-phosphate (G3P) as the most significantly differentially present and upregulated in IPD samples (Fig. 4d). G3P is known to be an intermediate metabolite in several metabolic pathways, including NAD metabolism, glycolysis, lipid metabolism and the electron shuttle between the cytoplasm and inner mitochondrial membrane (Fig. 4e). This means that G3P connects all metabolic pathways that we report as dysregulated in IPD NESCs, and therefore might be a valuable biomarker for IPD-associated early metabolic changes.

Furthermore, considering the essentiality of NAD as a cofactor being involved in all metabolic pathways dysregulated in IPD NESCs, we wanted to confirm that increasing NAD levels can rescue IPD NESC energy deficiency. Therefore, we treated cells with two different NAD precursors—quinolinic acid and nicotinic acid. We observed a significant increase in intracellular ATP levels in IPD NESCs, reaching ATP levels of the untreated control cells (Fig. 4f). These results further suggest that NAD deficiency might be the underlying cause of metabolic changes observed in IPD NESCs leading to the overall impairment of energy generation.

### Cross-match analysis with diagnosis-related metabolites

**Fig. 5:**
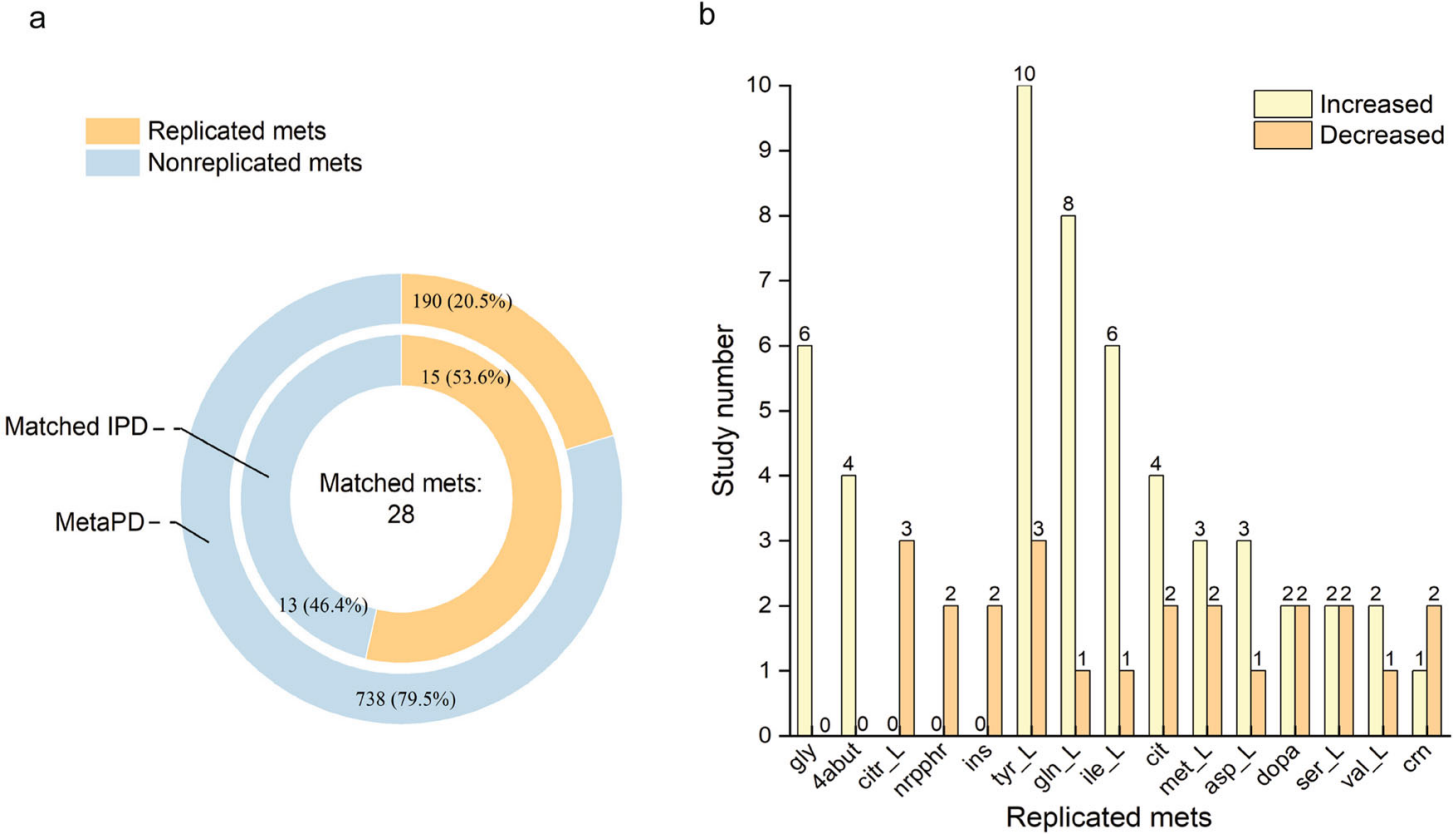
Metabolites corresponding to significant IPD model reactions compared with metabolites associated with PD diagnosis from a meta-analysis of clinical metabolomic studies. **a** The proportion of metabolites with changes reported in more than one study (replicated metabolite). **b** The number of studies reporting increased or decreased concentrations for replicated metabolites.

We wanted to confirm that the specific metabolic signature demonstrated in our work is consistent with PD prognostic metabolites reported in clinical metabolic studies. A meta-analysis of 74 clinical metabolic studies of PD by Fleming and colleagues revealed 928 metabolites associated with PD, of which only 21% (190/928) showed the same trend of qualitative change with respect to matched controls in more than one study^37^. We found that from 66 metabolites involved in the top 50 most differently behaving metabolic reactions in IPD metabolic models (Fig. 3a), 28 metabolites were associated with PD diagnosis matching 928 metabolites from the meta-analysis. 54% of those metabolites (15/28) were replicated in more than one clinical metabolomic study, matching 190 metabolites reported in the meta-analysis (Fig. 5a, b). The number of clinical studies reporting changes in each of these 15 replicated metabolites is shown in Fig. 5b and Table 1. Norepinephrine^38,39^ and citrulline^40–42^ were exclusively decreased in the plasma and serum of PD patients, respectively. Inosine was found decreased in both plasma^42^ and cerebrospinal fluid^43^ of PD patients, while glycine^38,44–48^ and GABA^38,45–47^ were increased in both plasma, urine and saliva of PD patients. Although the other 10 replicated metabolites showed an inconsistent concentration change in different studies, the levels of glutamine, aspartic acid, tyrosine, citric acid and isoleucine were significantly increased in the blood of PD patients in most of the studies. Methionine was identified as significantly changed in concentration over time in the plasma of PD patients^49^, while dopamine concentration change was observed in PD patient plasma, serum and CSF [35.39, 47, 48]. These results confirm that IPD has a complex pathogenesis, which is reflected in a specific metabolic signature and can be captured by a panel of metabolic biomarkers in various patient biospecimens. In this study, we show that NESCs can closely reproduce this specific signature.

**Table 1:** Summary of the matched replicated metabolites.

VMH ID	Common name	# Increased	# Decreased	Inconsistent	Increased samples	Decreased samples
nrpphr	Norepinephrine	0	2	0	–	Plasma^38,39^
dopa	Dopamine	2	2	1	Plasma^69^; CSF^70^	Plasma^38^; Serum^42^
ins	Inosine	0	2	0	–	Plasma^42^; CSF^43^
gly	Glycine	6	0	0	Plasma^38,48^; Serum^44^; Urine^45,46^; Saliva^47^	–
citr_L	Citrulline	0	3	0	–	Serum^40–42^
gln_L	Glutamine	8	1	1	Plasma^48,71,72^; Serum^44,73^; Putamen^74^; CSF^70^; Urine^75^	CSF^76^
asp_L	Aspartic acid	3	1	1	Plasma^38^; Urine^45^; Serum^77^	Serum^44^
ile_L	Isoleucine	6	1	1	Serum^73^; CSF^72^; Fecal^78^; Urine^45,75^; Saliva^47^	Fecal^79^
met_L	Methionine	3	2	1	Plasma^49,76^; CSF^70^	Plasma^49,80^
ser_L	Serine	2	2	1	Plasma^49^; CSF^76^	Serum^41,44^
tyr_L	Tyrosine	10	3	1	Plasma^39,48,81^; Serum^44,74,77,82^; Urine^45,75^; Saliva^47^	Fecal^79,83^; Plasma^84^
val_L	Valine	2	1	1	Serum^73^; Saliva^47^	Fecal^79^
4abut	GABA	4	0	0	Plasma^38^; Urine^45,46^; Saliva^47^	–
cit	Citric acid	4	2	1	Plasma^85^; Serum^73,86^; CSF^70^	Plasma^87^; Serum^44^
crn	Carnitine	1	2	1	Plasma^88^	Plasma^89^; CSF^70^

Metabolites identified in metabolic modeling to be changed in IPD cells and also associated with PD diagnosis in more than one clinical study. VMH ID: metabolite name in Recon 3D.

## Discussion

In the present study, we investigated primary metabolic changes in IPD patient-derived neural precursor cells. The transcriptomic data analysis confirmed significant metabolic alterations in IPD NESCs, specifically, in lipid, amino acid and pyruvate metabolism. Untargeted metabolomics suggested a particularly strong difference between IPD and control NESC lipid profiles. Along with metabolic pathways, many important biological processes were also significantly altered at the transcriptomics level, including cell cycle, RNA binding, regulation of membrane potential and neurodevelopment, emphasizing the tight crosstalk between metabolism, cellular functionality and developmental state.

We found that pyruvate metabolism is negatively regulated by the decreased expression of *LDHA*. Downregulation of *LDHA* in IPD NESCs might indicate the regulation of a metabolic shift, which is forcing pyruvate to enter the oxidative metabolism, and not be converted to lactate in order to continue anaerobic glycolysis. Additionally, *LDHA* has been described as a regulator of the cell cycle and proliferation state of cells^50^. Unrestricted neural stem cells initially rely on glycolysis; however, it has been shown that fate-restricted, mature progenitors, such as NESCs, undergo a metabolic shift, becoming dependent on oxidative metabolism^51–53^. However, we found that PDH activity was significantly decreased, suggesting an impairment of pyruvate involvement in TCA. Moreover, Seahorse extracellular flux analysis revealed that IPD NESCs have decreased respiratory capacity, mitochondrial ATP levels, and the ability to metabolize a variety of mitochondrial substrates. Further, we demonstrated significantly decreased NAD levels in IPD NESCs, which potentially is the primary cause of mitochondrial enzyme inactivity, since most of the reactions require NAD as a cofactor, including the PDH complex for pyruvate conversion to Acetyl-CoA. NAD has an essential role in cellular bioenergetics, mitochondrial health and cell survival^21,22,54^. Furthermore, NAD depletion has been observed in aging and neurodegenerative diseases, including PD^54–56^. Our results suggest that IPD neural progenitors might not be able to undergo the metabolic shift from glycolysis to oxidative metabolism due to the cofactor imbalance.

We complemented our analysis with metabolic modeling and data integration to have a deeper overview of genotype-phenotype relationships. We generated context-specific models applying media and bibliomic constraints using the XomicToModel pipeline^28^. Since in this study, we used NESCs that are precursors of midbrain dopaminergic neurons, bibliomic data specific for dopaminergic neuron metabolism was a great advantage and allowed us to investigate metabolic alterations relevant to dopaminergic neurons. For flux distribution prediction we used maximization of flux and concentration entropy, which was the best performing objective regards of flux prediction accuracy as reported by Preciat et al.^28^. Entropy is a measure of energy distribution in the system. Maximizing for entropy, the system tends toward a state at which the usage of energy is minimal. We observed that IPD metabolic models compared to control models showed mostly increased flux for the analyzed reactions. This might suggest that control models are already in an energetically favorable state, while IPD models present less energetically stable metabolic networks, and they have to increase fluxes through many metabolic pathways to reach an energetically optimal state. Metabolic shift accompanied by changes in entropy has been observed in many diseases and aging^57,58^. Furthermore, modeling confirmed a changed NAD metabolism in cytosolic reactions in IPD models. This was consistent with increased levels of G3P in IPD cells as G3P biosynthesis replenishes the cytosolic NAD pool to ensure the activity of metabolic pathways in the cytosol and allows energy generation via glycolysis. Since NAD cannot be transported between cytosol and mitochondria, both NAD pools are considered relatively independent but linked with the electron shuttles that allows regeneration of NAD^59,60^. We observed a decreased activity of mitochondrial respiration and impaired pyruvate oxidation by PDH, suggesting that particularly the mitochondrial NAD pool might be affected in IPD NESCs. Accordingly, treatment of NAD precursors increased the ATP generation capability of cells, allowing IPD NESCs to reach the ATP levels observed in control NESCs. Following this observation, we suggest that restoring NAD levels as early as possible might be an efficient preventative strategy for PD.

Using a data integration approach, we sought to identify a molecule that connects all altered metabolic pathways in IPD NESCs. All of the top discriminant gene transcripts showed a high degree of correlation with metabolites and Seahorse data, suggesting that the genetic signature plays an important role in disease development. However, in this analysis, we focused on metabolites since they might show higher specificity toward correlation with other features. We identified G3P as one of the highly correlating non-polar metabolites with genes and mitochondrial respiration features and the top significantly differentially abundant metabolite between IPD and control NESCs. Since we show that most dysregulated pathways in IPD NESCs include pyruvate metabolism, oxidative phosphorylation, NAD metabolism and lipid metabolism, G3P might have  a biomarker potential since it is at the crossroads of these pathways and therefore, could indicate their changed activity.

In summary, our results show that IPD NESCs undergo specific metabolic changes at early neurodevelopment and their metabolism seems to be less optimal in terms of energy distribution. Moreover, we confirm that a large part of metabolites involved in the predicted dysregulated metabolic reactions using the modeling approach was consistent with the metabolites reported with a concentration change in PD patient biological samples^37^. This suggests that predicted metabolic changes using metabolic modeling have a high degree of accuracy and are comparable to the clinical data. Additionally, we identify G3P as a metabolite connecting observed IPD-associated metabolic changes and NAD as a potential early disease-modifying agent. Overall, we show that experimental and computational methods can be combined to better elucidate molecular causes and associations of observed phenotypes. It was particularly interesting to see that it is possible to distinguish IPD samples from healthy control samples by using complex signatures.

There are some limitations of this study that have to be considered. This study was performed with a relatively small sample size (3 IPD vs 3 CTRL). Most of the experiments, particularly, untargeted metabolomics would benefit from a larger sample size, allowing better discrimination between the IPD and CTRL groups and an increase of statistical power. Furthermore, although we show decreased levels of NAD, we were not able to experimentally distinguish between cytosolic and mitochondrial NAD pools. However, we were able to separate mitochondrial and cytosolic metabolic NAD reactions in silico to estimate flux differences between the IPD and control NESCs. Nevertheless, modeling has rather a predictive value and for a definite result, all findings should be further validated experimentally. Non-polar or lipid metabolites were analyzed in an untargeted manner to achieve high coverage of metabolites and have a general overview of cellular metabolic profiles. In this case, most of these metabolites were not identified due to the limitations of the available library. Metabolites with no clear identity were not excluded from the study, however, they limit the interpretation of our data regarding specific lipid contributions to the disease phenotypes.

## Methods

### Ethical approval

The work with iPSCs has been approved by the Ethics Review Panel (ERP) of the University of Luxembourg and the national Luxembourgish Research Ethics Committee (CNER, Comité National d’Ethique de Recherche). CNER No. 201901/01; ivPD.

### Human neuroepithelial cell culture

NESCs were derived from iPSCs as described in ref. ^27^ from three female IPD patients and three female healthy controls (Supplementary Fig. 1a). NESC derivation from iPSC was achieved via embryonic body formation and expansion of neuroepithelium using small molecules CHIR99021 (CHIR, 3 μM) and purmorphamine (PMA, 0.5 μM). NESCs were cultured in N2B27 maintenance medium on 6-well plates (Thermo Scientific) pre-coated with Matrigel (Corning). N2B27 medium is composed of DMEM-F12 (Invitrogen) and Neurobasal (Invitrogen) 1:1 and supplemented with 1:200 N2 (Invitrogen), 1:100 B27 without vitamin A (Invitrogen), 1% Glutamax (ThermoFisher) and 1% penicillin/streptomycin (Invitrogen). For NESC maintenance, the N2B27 medium was freshly supplemented with small molecules—3 µM CHIR (Axon Medchem), 0.75 µM PMA (Enzo Life Science) and 150 µM ascorbic acid (Sigma). The medium was changed every second day and cells were routinely passaged at 80–90% confluence. Splitting was performed with Accutase (Sigma). Cells were kept in the incubator under constant conditions of 37 °C and 5% CO_2_. Cells were characterized by the expression of typical neural progenitor markers SOX2 1:100 (R&D Systems #BAF2018; RRID:AB_356217), PAX6 1:300 (Biolegend #901302; RRID:AB_2749901), Nestin 1:200 (BD Bioscience #611659; RRID:AB_399177) (Supplementary Fig. 1b).

### RNA sequencing and analysis

Total RNA was prepared using the Allprep DNA/RNA mini kit (QIAGEN #80204) following the supplier’s instructions. RNA-seq library was prepared from 1 μg of total RNA, using the NEBNext Ultra Directional RNA Library Prep Kit for Illumina (E7420L). Libraries were quantified with the KAPA Library Quantification Kit (Illumina GA/Universal, #KK4824). Samples were multiplexed and adjusted to 2 nM concentration, and 75-bp paired-end sequencing was performed by Nextseq 2000. Reads were mapped with Bowtie2 using hg19 as the reference genome. Gene differential expression analysis was performed in R (version 4.0.2) using the Biocundoctur package EdgeR with default parameters^61^. The significance for differential expression was adjusted for multiple hypothesis testing using the Benjamini and Hochberg method^62^. Gene enrichment analysis of significantly differential expressed genes (*p* < 0.05) was performed in R (version 4.0.2) using the Human Molecular Signatures Database (version 6.2.)^63,64^. Genes annotated to ‘Metabolic pathways’ were further assigned to specific pathways using the KEGG database^31^. The average log2FC was determined among all genes annotated in each pathway to determine the most dysregulated pathways.

### Derivatization and GC-MS measurement

Metabolite derivatization was performed by a multi-purpose sample preparation robot (Gerstel). Dried cell polar extracts were dissolved in 20 µl pyridine, containing 20 mg/ml methoxyamine hydrochloride (Sigma-Aldrich), for 90 min at 45 °C under shaking. After adding 20 µl N-methyl-N-trimethylsilyl-trifluoroacetamide (Macherey-Nagel), samples were incubated for 30 min at 45 °C under continuous shaking. Dried non-polar extracts were only dissolved in 30 µl N-methyl-N-trimethylsilyl-trifluoroacetamide and incubated for 60 min at 45 °C under continuous shaking.

GC-MS analysis was performed using an Agilent 7890B GC coupled to an Agilent 5977A inert XL Mass Selective Detector (Agilent Technologies). A sample volume of 1 µl was injected into a Split/Splitless inlet, operating in splitless mode at 270 °C. The gas chromatograph was equipped with a 30 m (I.D. 0.25 mm, film 0.25 µm) ZB-35ms capillary column (Phenomenex) with 5 m guard column in front of the analytical column. Helium was used as carrier gas with a constant flow rate of 1.2 ml/min. The GC oven temperature was held at 90 °C for 1 min and increased to 270 °C at 9 °C/min. Then, the temperature was increased to 320 °C at 25 °C/min and held for 7 min. The total run time was 30 min. The transfer line temperature was set to 280 °C. The MSD was operating under electron ionization at 70 eV. The MS source was held at 230 °C and the quadrupole at 150 °C. Mass spectra were acquired in full scan mode (m/z 70 to 700).

All GC-MS chromatograms were processed using MetaboliteDetector, v3.220190704^65^. Compounds were annotated by retention index and mass spectrum using an in-house mass spectral library. Retention index calibration was based on a C10–C40 even n-alkane mixture (Sigma-Aldrich). The internal standards, U-^13^C–Ribitol (Omicron Biochemicals), Pentanedioic-d_6_ acid (C/D/N Isotopes) and Tridecanoic-d_25_ acid (C/D/N Isotopes) were added at the same concentration to every sample to correct for uncontrolled sample losses and analyte degradation during metabolite extraction. The dataset was normalized using the response ratio of the integrated peak area analyte and the integrated peak area internal standard. Data were analyzed using RStudio (R version 4.0.2). Results were visualized using *tidyverse* package and *factoextra* package for the multivariate analysis.

### Metabolic microarray

Mitochondria substrate preferences were assessed using MitoPlate S-1 (Biolog) following the manufacturer’s instructions. The MitoPlate S-1 assay is a colorimetric assay and measurement is based on the electron flow rate through the mitochondrial respiratory chain. Tetrazolium redox dye MC acts as the final electron acceptor changing a color proportional to the rate of substrate metabolization, which is recorded as Omnilog units. 96-well microplate MitoPlate S-1 is pre-coated with 31 different NADH and FADH2- producing metabolic substrates. Plate layout allows measurements in triplicates, therefore each IPD and control cell line was seeded in 32 wells of the plate (31 substrates + blank). Before cell seeding, substrates were incubated for 1 h at 37 °C with the assay mix containing assay buffer, redox dye, water and permeabilization reagent saponin (100 µg/ml per well). Then, 20,000 cells were seeded per well and directly loaded in OmnilogLog (Biolog) for kinetic reading every 5 min for 10 h. After measurement, plates were centrifuged and cell density was measured with CyQUANT Cell Proliferation Assay (Invitrogen). CyQUANT GR solution was prepared following the kit protocol. Fluorescence was measured at 520 nm on a cell imaging system Cytation5M. Kinetic data was analyzed with Data Analysis software (version 1.7.). The maximal metabolic rate for each substrate between the initial and 8 h measurements was calculated by background subtraction and normalization to cell density in the respective well. Negative values were replaced with 0. A median of the normalized maximal rate between three IPD and three control samples for each substrate was visualized as a scatter plot using RStudio (R version 4.0.2).

### Cellular metabolism analysis

A day before the experiment, the XFe96 sensor cartridge (Agilent) was filled with Seahorse XF Calibrant Solution (Agilent) (200 µl per well) and kept at 37 °C overnight. Before the experiment, the Seahorse XFe96 Spheroid Microplate (Agilent) was coated with Cell-Tak (Corning) coating diluted in 0.2 M Sodium bicarbonate solution (1:50) (coating 30 µl per well). After 1 h of incubation at 37 °C, the plate was washed twice with filtered MiliQ. 100 000 were cells seeded per well; each cell line in 8 replicates (wells). To ensure the complete attachment of cells, the plate was incubated at 37 °C for 3–4 h. After the incubation time, cells were washed twice with Seahorse media (for Mito stress test: DMEM Sigma D5030 + 21.25 mM glucose and 1 mM pyruvate; for glycolysis stress test: DMEM Sigma D5030 without glucose and pyruvate). All wells were filled with 180 µl of Seahorse media and incubated for 1 h at 37 °C. For Mito Stress analysis XFe96 sensor cartridge was filled with drugs as follows: Port A: Oligomycin 1 µM, Port B: FCCP 1 µM, and Port C: Antimycin 1 µM + Rotenone 1 µM (1:1). For the Glycolysis stress test XFe96 sensor cartridge was filled with drugs as follows: Port A: Glucose 10 mM, Port B: Oligomycin 1 µM, Port C: 2-deoxyglucose (2DG) 50 mM. Analysis was performed using the XFe96 Cell Metabolism Analyzer. The obtained data was normalized to the cell density measured with CyQUANT Cell Proliferation Assay (Invitrogen). CyQUANT GR solution was prepared following the kit protocol. Then, 200 µl of the solution was added to each well of the Seahorse microplate (including empty wells). After 2 min of incubation, the content of the microplate was transferred to the imaging microplate (Perkin Elmer) compatible with the available cell imaging system Cytation5M. Fluorescence was measured at 520 nm.

Both assays were performed three times using three different cell passages. Each well of the assay was represented as a separate data point. Wells with negative values or aberrant OCR or ECAR patterns were excluded from each of the individual experiments. Data was analyzed with GraphPad Prism version 9.

### Western blot

Cells were collected at 80% confluency and lysed using RIPA buffer (Abcam). Protein was quantified using BCA assay (ThermoFisher). Then, 20 µg of protein was loaded per sample in 4–12% NuPAGE Bis-Tris polyacrylamide gel (ThermoFisher). Gel was run using MES SDS running buffer (ThermoFisher), following dry transfer on polyvinylidene difluoride membrane using iBlot (ThermoFisher). The membrane was blocked for 1 h in 5% BSA in PBS + 0.02% Tween solution following overnight incubation with primary antibody for PDHA1 1:500 (Cell Signaling Technology #3205; RRID:AB_2162926). The membrane was washed 3 times for 5 min with PBS + 0.02% Tween. After washing membrane was incubated for 1 h with a secondary anti-rabbit, HRP-linked antibody (VWR). After 3 washing steps for 10 min, a signal was developed using a chemiluminescent substrate (Life Technologies) and imaged with STELLA 8300 imaging system (Raytest). After the reveal of the PDHA1 protein signal, the membrane was stripped for 15 min with a stripping buffer (ThemoFisher) to remove the signal. The membrane was washed 3 times for 10 min following blocking and overnight incubation with β-actin primary antibody 1:10,000 (Cell Signaling Technology #3700, RRID:AB_2242334). β-actin protein was analyzed following the same procedure as described for PDHA1 protein. The experiment was performed three times for different NESC passages. Band intensity was quantified using ImageJ and PDHA1 relative abundance was estimated by normalizing the PDHA1 protein signal to the β-actin signal. Original blot images of one of the experiments are shown in Supplementary Fig. 7.

### ATP assay

Intracellular ATP was measured using CellTiter-Glo® Luminescent Cell Viability Assay (Promega). Cells from three different passages were seeded in a Geltrex (ThermoFisher) pre-coated 96-well imaging plate (Perkin Elmer). The next day after seeding, cells were treated with 20 nM quinolinic acid (Sigma #P63204), 5 mM nicotinic acid (Sigma #N0761) or ultra-pure water as vehicle. After 24 h, the media was replaced by 50 µl of ATP assay reagent. After 10 min of incubation in the dark, luminescence was read with the cell imaging system Cytation5M. After luminescence recording, the ATP assay reagent was removed and the cell count in each well was determined using CyQUANT Cell Proliferation Assay (Invitrogen). The luminescence signal of ATP was normalized to the cell count.

### Pyruvate dehydrogenase activity assay

Pyruvate dehydrogenase activity was detected using the PDH activity assay kit (Sigma MAK183) following the manufacturer’s instructions. Kit measures generated NADH amount which is proportional to enzyme activity. One million cells were pelleted and then resuspended in an ice-cold PDH assay buffer. After 10 min of incubation on ice, samples were centrifuged at 10,000×*g* for 5 min. Then, 10 µl of supernatant in duplicates were collected in a 96-well flat bottom plate (Corning) for each sample and mixed by shaking with the PDH assay substrate and developer. Samples were then incubated at 37 °C. Absorbance at 450 nm was measured after 3–5 min of incubation, following further incubation at 37 °C. The absorbance reading was repeated until the highest sample values reached the linear range of the standard curve. PDH activity was calculated as NADH amount in nmol generated between the initial and final absorbance reading. The assay was repeated three times for three different NESC passages. The results of each run were normalized to the mean of control samples of the respective run. Data were visualized using GraphPad Prism version 9.

### NAD+/NADH quantification

The NAD+/NADH ratio was measured with a Sigma kit (MAK037) following the manufacturer’s instructions. One million cells were pelleted by centrifugation. After the removal of the media, the pellet was washed with cold PBS. Total NAD+ and NADH were extracted by cell incubation with extraction buffer during two freezing–thawing cycles of sample incubation in dry ice for 20 min following incubation at room temperature for 10 min. Samples were then vortexed and centrifuged to remove insoluble material. For detection of NADH only, samples were further incubated at 60 °C for 3 min and cooled on ice afterwards. The concentration of the total NAD and NADH were calculated using the NADH standard curve. The NAD+/NADH ratio was estimated by subtracting NADH concentration from the total NAD and dividing by NADH. Each experiment was normalized to the median of the control sample results. The assay was repeated three times for three different NESC passages.

### Constraint-based metabolic modeling

For each IPD patient-derived and each control NESC line, a separate metabolic model (IPD1, IPD2, IPD3 and CTRL1, CTRL2, CTRL3) was generated. Model extraction was performed using the XomicsToModel pipeline^28^ within the COBRA Toolbox v3.4^66^ on MATLAB version 2021b.

Context-specific models were extracted using:

1. The thermodynamic, stoichiometrically and flux consistent subset of Recon 3D as a generic model, a genome-scale model for human metabolism.
2. Context-specific data from transcriptomic and cell culture data. Normalized counts to Transcripts Per Kilobase Million (TPM) were transformed to log2 scale. The genes above a selected threshold were considered active and included in the model. Additionally, cell culture data provided the uptakes for each of the models based on Eq. 1 where cell DW for a single cell was calculated considering that the total protein fraction within the dopaminergic neurons is 55.93%^67^ and protein concentration in each NESC line was determined using BCA kit (ThermoFisher).
  1 $${uptake}=\frac{{metabolite}\,{concentration}\,({{{{{\rm{\mu }}}}}}{mol}/L)\times {media}\,{volume}\,(L)}{{cell}\,{dry}\,{weight}\,\left({gDW}\right)\times {protein}\,{concentration}\times {interval}\,({hr})}$$
3. Literature curation on dopaminergic neuron metabolism including dopamine metabolism, mitochondrial and central carbon metabolism, biomass precursors and media metabolites, presented in ref. ^29^ for setting reaction rates, and identifying active and inactive reactions, metabolites and genes. The literature curation used in ref. ^29^ was modified by excluding genes whose expression in the brain was referred only to animal models.
4. Same XomicsToModel parameters were used for the generation of the iDopaNeuro model^29^ including *thermoKernel* as the tissue-specific solver; at least one active reaction per active gene as the method includes reactions corresponding to active genes, two TPM mapped reads as the transcriptomic threshold, below which genes were designated as inactive, closing the ion exchange and prioritize experimental data over literature curation.

Flux distribution was determined by eFBA simultaneously maximizing the entropy of forward and reverse flux with the objective shown in Eq. (2) using the Mosek version 9.3.20 entropic programming solver.

2 $${\varPsi }_{v}={v}_{f}^{T}\, {{{{\mathrm{ln}}}}}{v}_{f}+{v}_{r}^{T}\, {{{{\mathrm{ln}}}}}{v}_{r}$$

Where ${\varPsi }_{v}$ represent the objective, $v$ the fluxes in ${\mu mol}/{gDW}/{hr}$ and the subindices $f$ and $r$ indicate forward and reverse, respectively. The fold change was calculated between shared reactions in all models based on Eq. (3)

3 $${fold}\,{change}=\frac{{reaction}\,{flux}-{control}\,{mean}\,{flux}}{{control}\,{mean}\,{flux}}$$

### Data integration analysis

Experimental datasets of TPM normalized RNA expression, polar-phase and non-polar-phase metabolites from untargeted GC-MC analysis and mitochondrial phenotypes from Seahorse mitochondrial metabolism assay were integrated using Data Integration Analysis for Biomarker discovery using latent components (DIABLO)^68^ within the R software (version 4.0.2.). DIABLO aims at using combined metabolomic, transcriptomic and phenotypic data to separate healthy from IPD NESCs. The separation between sample groups was assessed using the correlation of the top 50 genes with the top 10 metabolites per dataset and 6 mitochondrial phenotypes from the first component of PLS-DA^68^. Heatmap visualization was used to demonstrate the top feature (gene, metabolite and mitochondrial phenotype) expression profile responsible for cluster separation between conditions. Pearson correlations (abs(r) = 0.9) between features were further visualized in a circus plot. Features with the highest correlation degree (abs(r) = 0.95) are represented as a network using Cytoscape (version 3.8.2.).

### Statistics and reproducibility

All experiments were performed with all three cell lines per condition representing biologically independent samples. All experiments were independently repeated three times using different cell passages. Except for RNA sequencing and metabolomics, which were repeated once. For statistical analysis, GraphPad Prism version 9 was used. Data were tested for outliers with the ROUT method (Q = 1%) and for normality with the Shapiro-Wilk test. For data of non-normal distribution statistical significance was determined by a two-tailed non-parametric Mann-Whitney test. For data of normal distribution, statistical significance was determined by unpaired two-tailed *t*-test if not stated differently. Significance asterisks represent *P < 0.05, **P < 0.01, ***P < 0.001, ****P < 0.0001. Error bars represent mean + SD.

### Reporting summary

Further information on research design is available in the Nature Portfolio Reporting Summary linked to this article.

### Supplementary information

Supplementary Information

Description of Additional Supplementary Files

Supplementary data 1

Supplementary data 2

Supplementary data 3

Supplementary data 4

Supplementary data 5

Reporting Summary

## Data Availability

All related data supporting the findings of this study are publicly available at 10.17881/v8jg-pw83. RNA sequencing data are deposited to the Gene Expression Omnibus database under accession number: GSE207088. Metabolomics data are deposited to the EMBL-EBI MetaboLights database with the identifier MTBLS7740.

## Code availability

All custom Matlab code for metabolic model generation and R scripts for data analysis and visualization are available on GitHub: https://gitlab.lcsb.uni.lu/dvb/zagare_2022.
