# Supplementary material for: Omics data integration suggests a potential idiopathic Parkinson’s disease signature
Source: Commun Biol. 2023 Nov 20;6:1179. doi: 10.1038/s42003-023-05548-w (PMC10662437; doi:10.1038/s42003-023-05548-w)
Supplement: Supplementary file 1 — Supplementary Information [file 42003_2023_5548_MOESM1_ESM.pdf]

a

Source of IPSCs	IPSC ID	Disease	Age at onset	Age at sampling	Sex	Corresponding NESC line
IBBL/MPI Muenster	2.0.0.70.2.0	WT	-	65	Female	CTRL1
IBBL/MPI Muenster	2.0.0.72.1.0	WT	-	63	Female	CTRL2
IBBL/MPI Muenster	2.0.0.71.1.0	WT	-	68	Female	CTRL3
IBBL/MPI Muenster	2.1.0.67.3.0	IPD	57	67	Female	IPD1
IBBL/MPI Muenster	2.1.0.68.2.0	IPD	62	67	Female	IPD2
IBBL/MPI Muenster	2.1.0.69.5.0	IPD	60	68	Female	IPD3

b

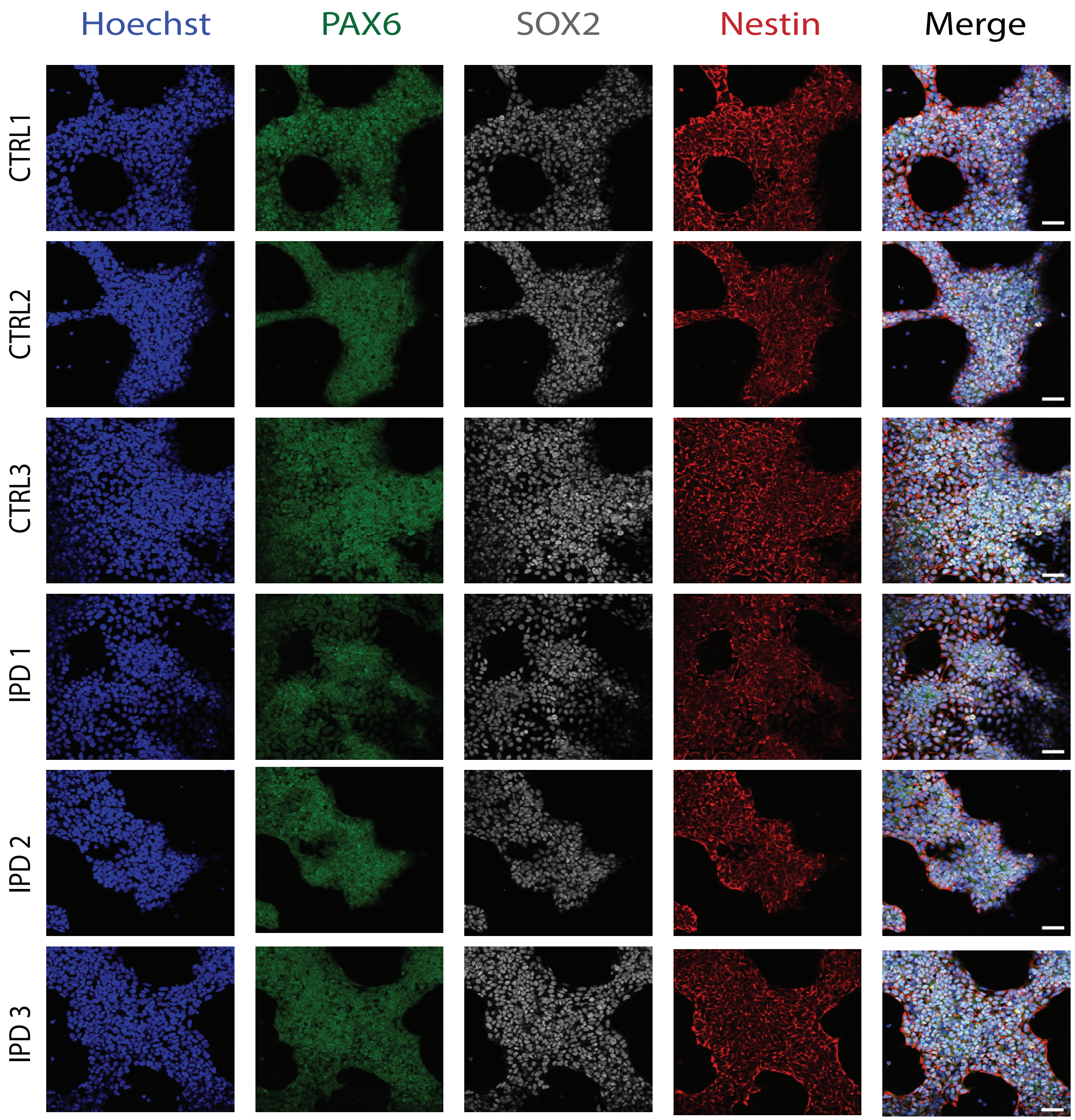

Supplementary Fig. 1. .Cell lines used in this study. a) Source of IPSC used to generate NESCs. IBBL: Integrated Biobank of Luxembourg. MPI Muenster: Max Planck Institute in Muenster. b) NESC characterization by iimmunofluorescent stainings SOX2 (grey), PAX6 (green), Nestin (red). Images acquired with 20x objective, scale bars: 50µm.

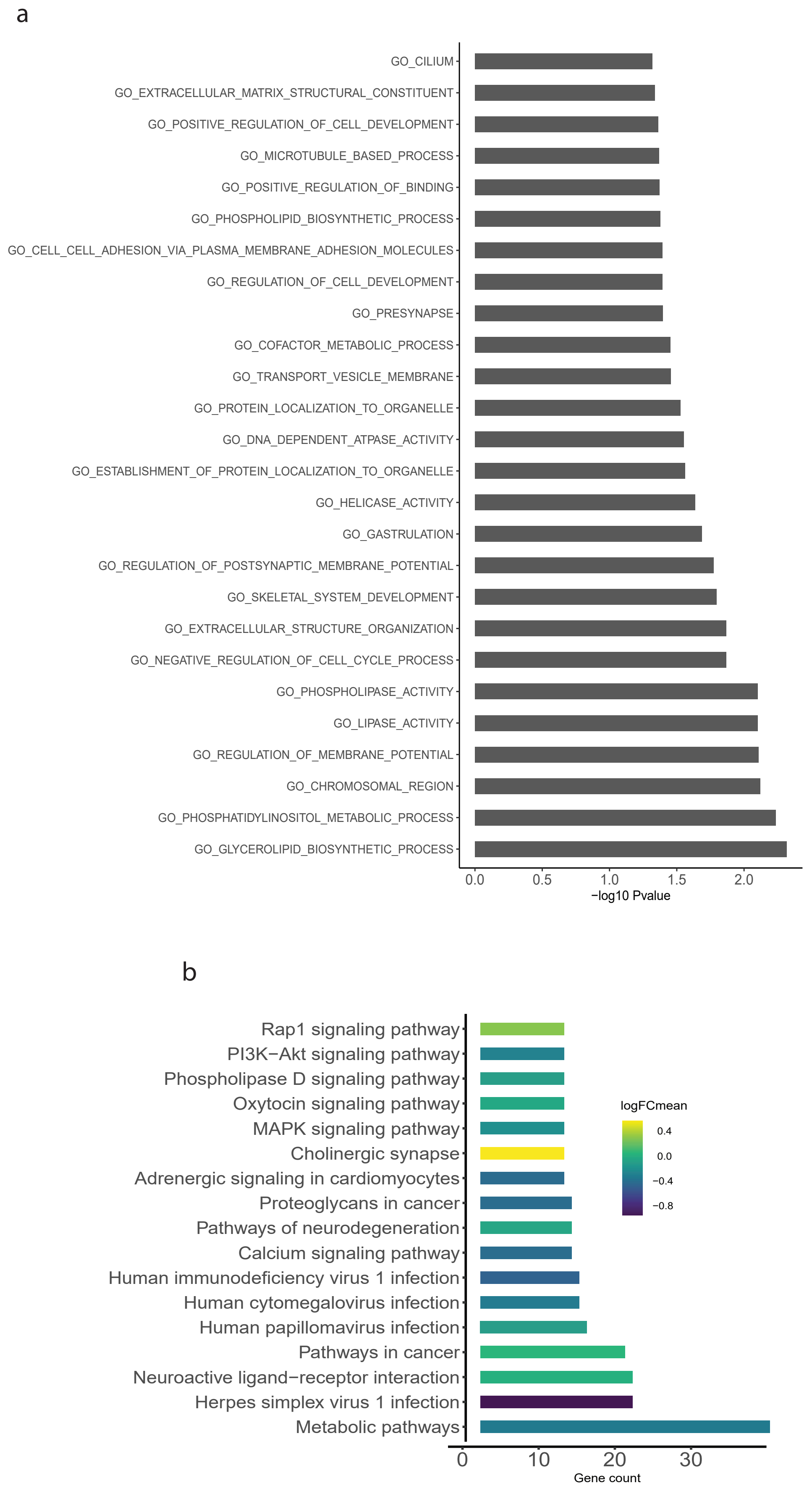

Supplementary Fig. 2. Transcriptomic and metabolic profiles reveal neurodevelopmental and metabolic alterations in IPD neural precursor cells. a) The most significantly enriched GO terms ($p<0.05$) of DEGs. b) DEG annotation to the KEGG database. The color indicates the average log2FC of the respective genes.

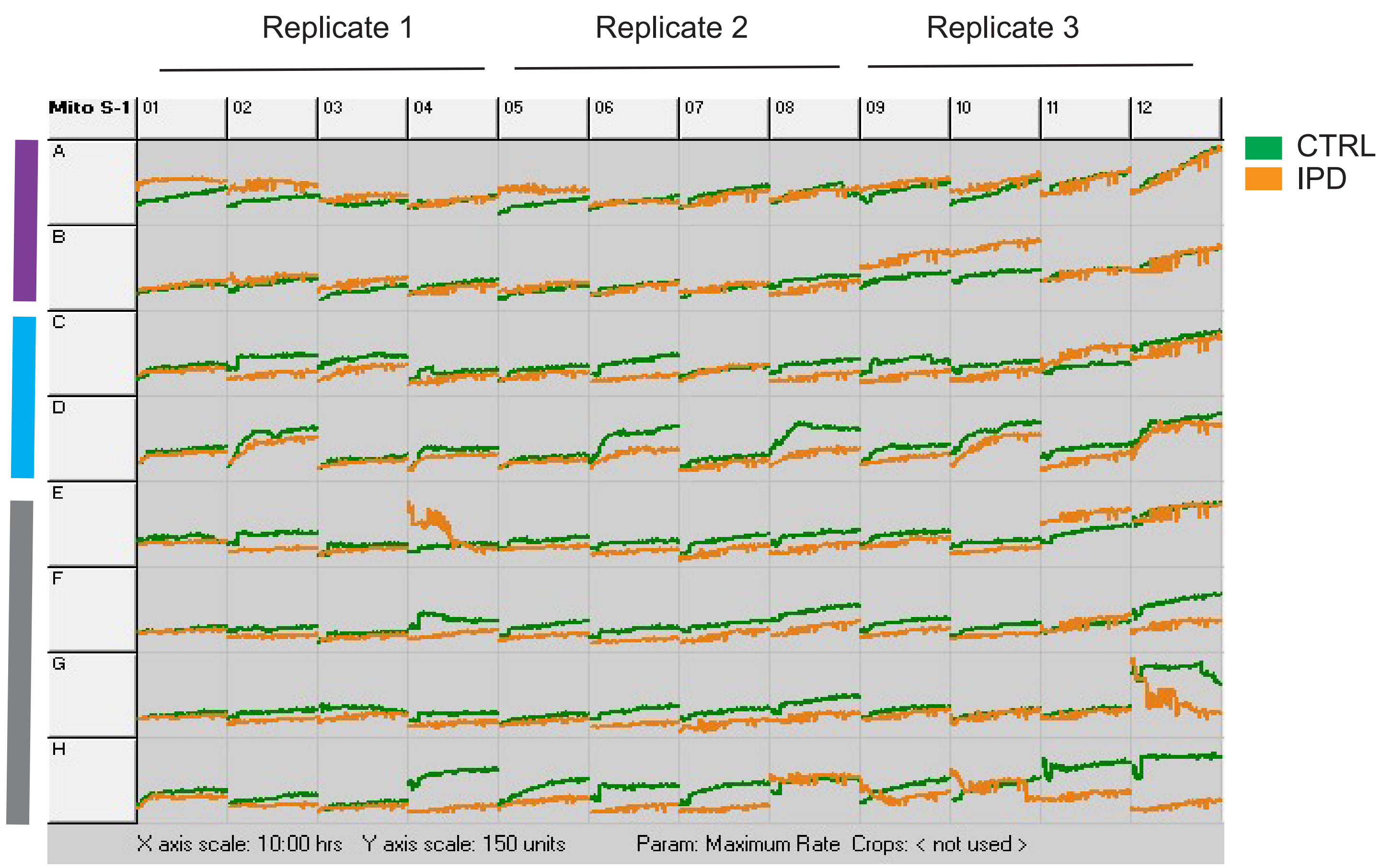

A1; A5; A9: no substrate/blank

Cytoplasmic substrates

A2; A6; A10: α-D-Glucose
A3; A7; A11: Glycogen
A4; A8; A12: D-Glucose-1-PO4
B1; B4; B9: D-Glucose-6-PO4
B2; B6; B10: D-gluconate-6-PO4
B3; B7; B11: D,L-alpha-Glycerol-PO4
B4; B8; B12: L-Lactic Acid

TCA cycle substrates

C1; C5; C9: Pyruvic acid
C2; C6; C10: Citric Acid
C3; C7; C11: D,L-Isocitric Acid
C4; C8; C12: cis-Aconitic Acid
D1; D5; D9: α-Keto-Glutaric Acid
D2; D6; D10: Succnic Acid
D3; D7; D11: Fumaric Acid
D4; D8; D12: L-Malic Acid

Other mitochondrial substrates

E1; E5; E9: α-Keto-Butyric Acid
E2; E6; E10: D,L-β-Hydroxy Butyric Acid
E3; E7; E11: L-Glutamic Acid
E4; E8; E12: L-Glutamine
F1; F5; F9: Analyl-Glutamine
F2; F6; F10: L-Serine
F3; F7; F11: L-Ornithine
F4; F8; F12: Tryptamine

G1; G5; G9: 100μM L-Malic Acid
G2; G6; G10: Acetyl L-Carnitine + 100μM L-Malic Acid
G3; G7; G11: Octanoyl-L-Carnitine + 100 μM L-Malic Acid
G4; G8; G12: Palmitoyl-D,L-Carnitine + 100 μM L-Malic Acid
H1; H5; H9: Pyruvic Acid + 100 μM L-Malic Acid
H2; H6; H10: γ-Amino-Butyric Acid + 100 μM L-Malic Acid
H3; H7; H11: α-Keto-Isocaproic Acid + 100 μM L-Malic Acid
H4; H8; H12: L-Leucine + 100 μM L-Malic Acid

Supplementary Fig. 3. The layout of MitoPlate S1. The raw kinetic curves exported of Data Analysis software 1.7. and the metabolic substrate types present in the microplate.

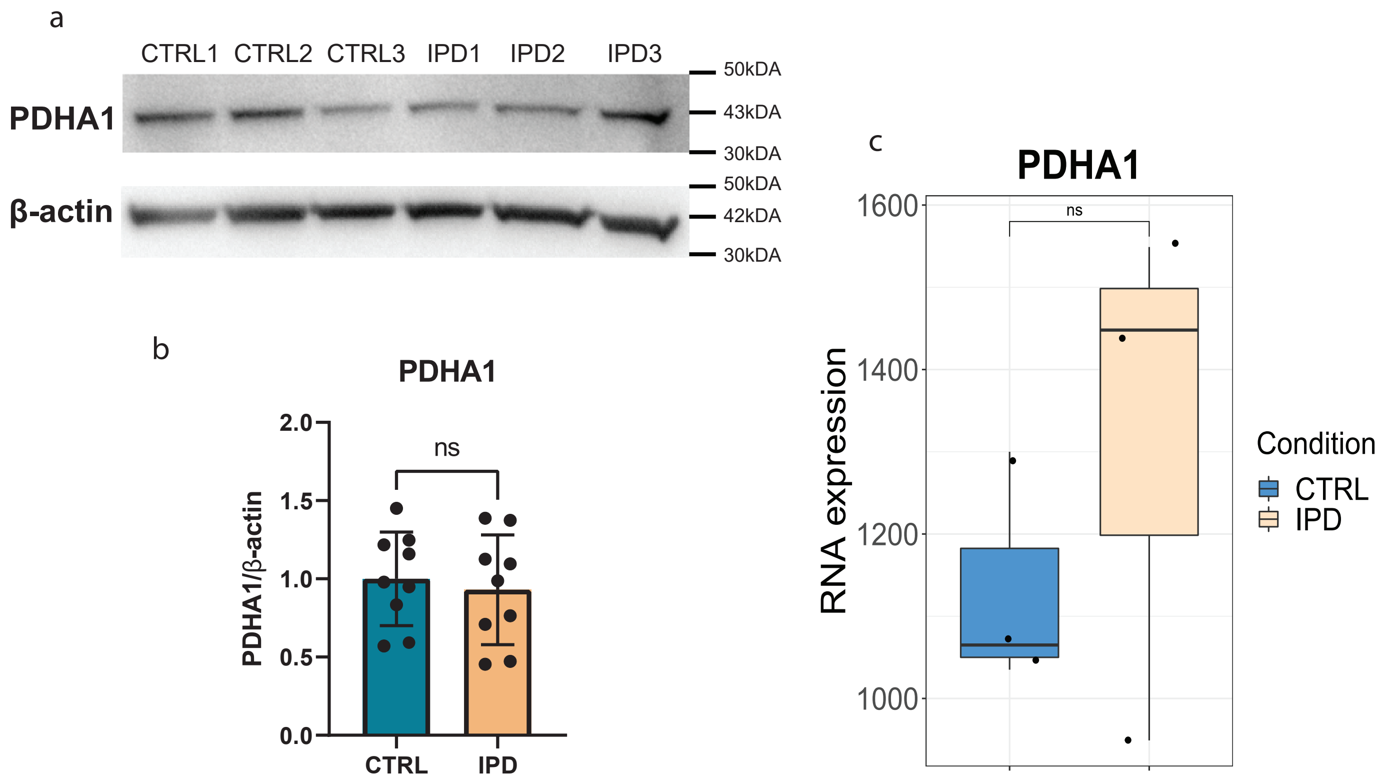

Supplementary Fig. 4. Quantification of PDHA1. a) A representative Western blot showing the expression of PDHA1 and β-actin. b) Quantification of protein expression detected by Western blot. Relative expression of PDHA1 normalized to the mean expression in control lines within each experiment (N=3). Error bars represent mean + SD. c) RNA expression of *PDHA1* from RNA sequencing analysis.

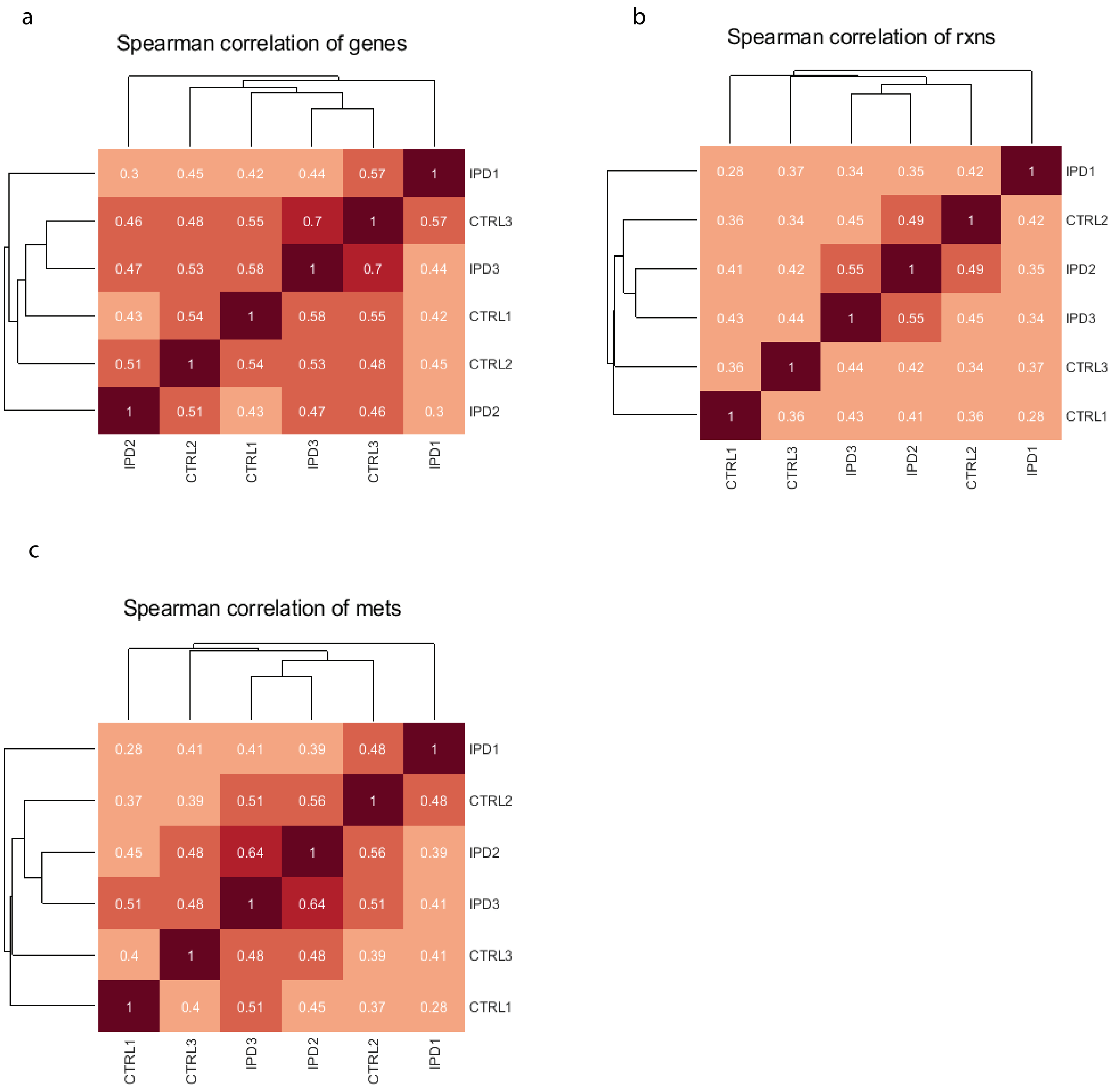

Supplementary Fig. 5. Metabolic modeling. a) Spearman correlation of genes present in the generated models. b) Spearman correlation of reactions present in the generated models. c) Spearman correlation of metabolites present in the generated models.

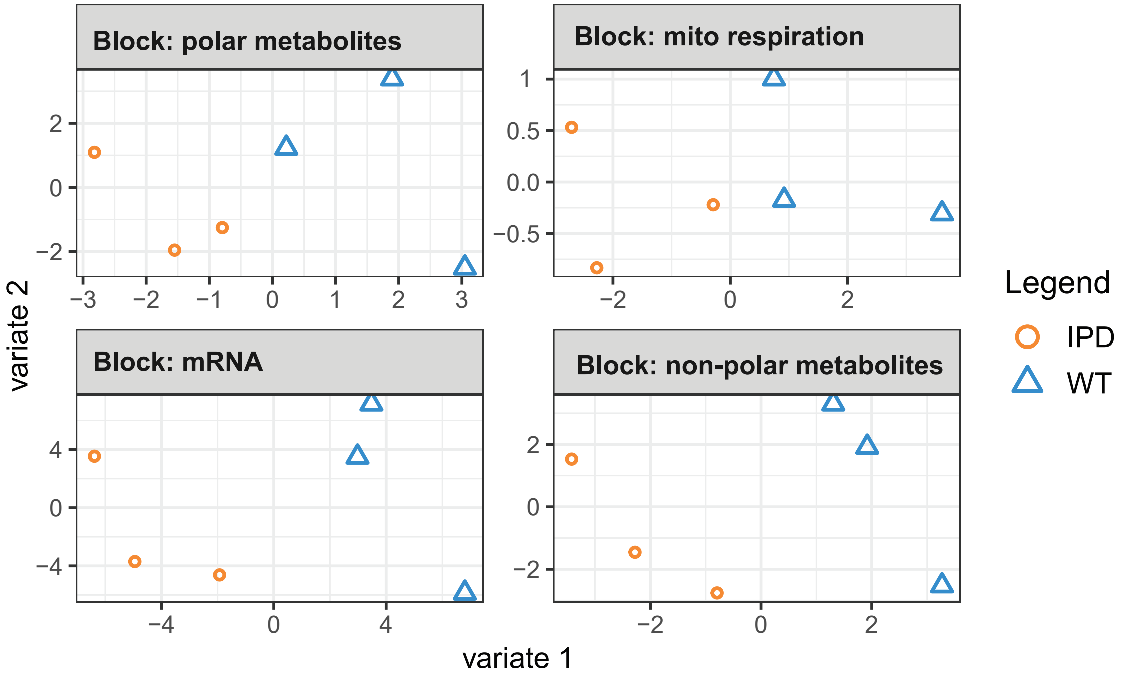

Supplementary Fig. 6. Integration analysis. Discriminant analysis of single datasets based on two dimensions (variate 1 and variate 2) of the top 10 polar and non-polar metabolites, top 50 genes and 6 mitochondrial respiration features.

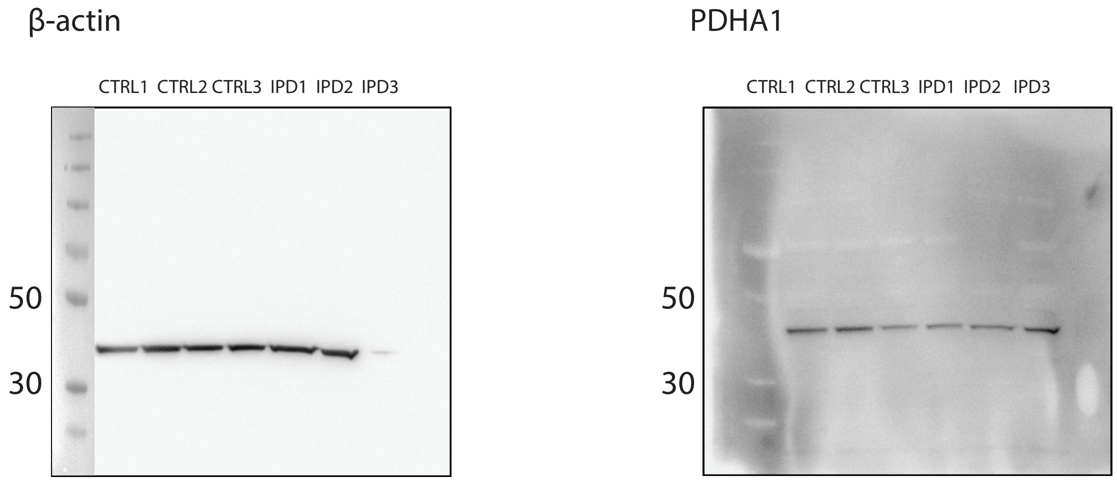

Supplementary Fig. 7. Western Blot scans for Supplementary Fig. 4 showing the protein abundance of β-actin and PDHA1. Size marke included for β-actin, since it was not visible on the membrane with the protein.
