# Supplementary material for: Omics data integration suggests a potential idiopathic Parkinson’s disease signature
Source: Commun Biol. 2023 Nov 20;6:1179. doi: 10.1038/s42003-023-05548-w (PMC10662437; doi:10.1038/s42003-023-05548-w)
Supplement: Supplementary file 2 — Description of Additional Supplementary Files [file 42003_2023_5548_MOESM2_ESM.pdf]

**Description of Additional Supplementary Files**

**File name:** Supplementary Data 1
**Description:** Differentially expressed genes between IPD and CTRL

**File name:** Supplementary Data 2
**Description:** The top 50 metabolic reactions with the highest flux change between IPD and CTRL

**File name:** Supplementary Data 3
**Description:** Metabolic reactions involved in NAD metabolism

**File name:** Supplementary Data 4
**Description:** Mitochondrial reactions involving NAD

**File name:** Supplementary Data 5
**Description:** Cytosolic reactions involving NAD
