# Supplementary material for: Omics data integration suggests a potential idiopathic Parkinson’s disease signature
Source: Commun Biol. 2023 Nov 20;6:1179. doi: 10.1038/s42003-023-05548-w (PMC10662437; doi:10.1038/s42003-023-05548-w)
Supplement: Supplementary file 8 — Reporting Summary [file 42003_2023_5548_MOESM8_ESM.pdf]

nature portfolio

Corresponding author(s): Jens Christian Schwamborn

Last updated by author(s): 2023/10/23

Reporting Summary

Nature Portfolio wishes to improve the reproducibility of the work that we publish. This form provides structure for consistency and transparency in reporting. For further information on Nature Portfolio policies, see our Editorial Policies and the Editorial Policy Checklist.

Statistics

For all statistical analyses, confirm that the following items are present in the figure legend, table legend, main text, or Methods section.

n/a	Confirmed	
☐	☒	The exact sample size (*n*) for each experimental group/condition, given as a discrete number and unit of measurement
☐	☒	A statement on whether measurements were taken from distinct samples or whether the same sample was measured repeatedly
☐	☒	The statistical test(s) used AND whether they are one- or two-sided<br>*Only common tests should be described solely by name; describe more complex techniques in the Methods section.*
☒	☐	A description of all covariates tested
☐	☒	A description of any assumptions or corrections, such as tests of normality and adjustment for multiple comparisons
☒	☐	A full description of the statistical parameters including central tendency (e.g. means) or other basic estimates (e.g. regression coefficient) AND variation (e.g. standard deviation) or associated estimates of uncertainty (e.g. confidence intervals)
☒	☐	For null hypothesis testing, the test statistic (e.g. *F*, *t*, *r*) with confidence intervals, effect sizes, degrees of freedom and *P* value noted<br>*Give P values as exact values whenever suitable.*
☒	☐	For Bayesian analysis, information on the choice of priors and Markov chain Monte Carlo settings
☒	☐	For hierarchical and complex designs, identification of the appropriate level for tests and full reporting of outcomes
☒	☐	Estimates of effect sizes (e.g. Cohen's *d*, Pearson's *r*), indicating how they were calculated

Our web collection on statistics for biologists contains articles on many of the points above.

Software and code

Policy information about availability of computer code

Data collection	No unpublished software was used for data collection
Data analysis	Data analysis was done with custom code using Cobra toolbox in MATLAB version 2021b, data analysis with GraphPad Prism 9, data analysis with RStudio (R version 4.0.2).

For manuscripts utilizing custom algorithms or software that are central to the research but not yet described in published literature, software must be made available to editors and reviewers. We strongly encourage code deposition in a community repository (e.g. GitHub). See the Nature Portfolio guidelines for submitting code & software for further information.

Data

Policy information about availability of data

All manuscripts must include a data availability statement. This statement should provide the following information, where applicable:

- Accession codes, unique identifiers, or web links for publicly available datasets
- A description of any restrictions on data availability
- For clinical datasets or third party data, please ensure that the statement adheres to our policy

Data availability
All related data supporting the findings of this study is publicly available at DOI: https://doi.org/10.17881/v8jg-pw83. RNA sequencing data are deposited to the Gene Expression Omnibus database under accession number: GSE207088. Metabolomics data are deposited to the EMBL-EBI MetaboLights database with the

identifier MTBLS7740.

Code availability
All custom Matlab code for metabolic model generation and R scripts for data analysis and visualization are available on GitHub: https://gitlab.lcsb.uni.lu/dvb/zagare_2022.

Research involving human participants, their data, or biological material

Policy information about studies with human participants or human data. See also policy information about sex, gender (identity/presentation), and sexual orientation and race, ethnicity and racism.

Reporting on sex and gender	In this study we used human induced-pluripotent stem cell derived neuroepithelial stem cells from three female idiopathic Parkinson disease patients and age-gender matched healty individuals.
Reporting on race, ethnicity, or other socially relevant groupings	NA
Population characteristics	NA
Recruitment	NA
Ethics oversight	The work with iPSCs has been approved by the Ethics Review Panel (ERP) of the University of Luxembourg and the national Luxembourgish research ethics committee (CNER, Comité National d'Ethique de Recherche). CNER No. 201901/01; ivPD

Note that full information on the approval of the study protocol must also be provided in the manuscript.

Field-specific reporting

Please select the one below that is the best fit for your research. If you are not sure, read the appropriate sections before making your selection.

☒ Life sciences ☐ Behavioural & social sciences ☐ Ecological, evolutionary & environmental sciences

For a reference copy of the document with all sections, see nature.com/documents/nr-reporting-summary-flat.pdf

Life sciences study design

All studies must disclose on these points even when the disclosure is negative.

Sample size	Sample size was based on the cell line availability. Three patient vs three healthy control cell lines
Data exclusions	Data was excluded based on outlier test ROUT. In the Seahorse mitochondrial assay data was excluded in case of failed response to the drugs.
Replication	Metabolomics and RNA sequencing was performed once. Metabolomics included technical triplicates for each sample. All other experiments were performed at least from three different cell passages.
Randomization	Three patient-derived cell lines were compared to three healthy individual derived cell lines.
Blinding	Metabolomics, RNA sequencing and model analysis was performed using sample identifiers that do not represent the healthy or patient group. In other experiments samples were not blinded

Reporting for specific materials, systems and methods

We require information from authors about some types of materials, experimental systems and methods used in many studies. Here, indicate whether each material, system or method listed is relevant to your study. If you are not sure if a list item applies to your research, read the appropriate section before selecting a response.

Materials & experimental systems

n/a	Involved in the study
☐	☒ Antibodies
☐	☒ Eukaryotic cell lines
☒	☐ Palaeontology and archaeology
☒	☐ Animals and other organisms
☒	☐ Clinical data
☒	☐ Dual use research of concern
☒	☐ Plants

Methods

n/a	Involved in the study
☒	☐ ChIP-seq
☒	☐ Flow cytometry
☒	☐ MRI-based neuroimaging

Antibodies

Antibodies used

Pyruvate dehydrogenase rabbit (Cell Signaling Technology #3205; RRID:AB_2162926)
β-actin mouse primary antibody (Cell Signaling Technology #3700, RRID:AB_2242334)
SOX2 (R&D Systems #BAF2018; RRID:AB_356217)
PAX6 (Biolegend #901302; RRID: AB_2749901)
Nestin (BD Bioscience #611659; RRID:AB_399177)

Validation

Pyruvate dehydrogenase: UniProt ID: P08559, validated in various cell lines, reference: Sargsyan et al., 2023, JCI Insight. doi:10.1172/jci.insight.153740.
β-actin: UniProt ID: P60709, validated in various cell lines, reference: Kiseleva et al., 2023, Nucleus.doi: 10.1080/19491034.2023.2165602.
SOX2: validated in human cells, reference: Graham, V. et al. (2003) Neuron 39:749.
PAX6: immunohistochemical staining validated in brain tissue, reference: Ahmad Z, et al. 2015. PLoS One. doi:1https://doi.org/10.1371/journal.pone.0144597.
Nestin: Knockout validation, reference: Cui et al., Nat Commun. 2016;7:1063

Eukaryotic cell lines

Policy information about cell lines and Sex and Gender in Research

Cell line source(s)

The source of cell lines used in the study is Integrated Biobank of Luxembourg in collaboration with Max Planck Institute in Muenster. Sex:female

Authentication

Cell lines have been karyotyped

Mycoplasma contamination

Cell lines are tested negative for Mycoplasma. Or lab performs routinely Mycoplasma tests using LookOut® Mycoplasma PCR Detection Kit (Sigma, cat.no. MP0035-1KT).

Commonly misidentified lines
(See ICLAC register)

NA
